# Functional Genomic Screening in Human Pluripotent Stem Cells Reveals New Roadblocks in Early Pancreatic Endoderm Formation

**DOI:** 10.3390/cells11030582

**Published:** 2022-02-08

**Authors:** Jana Krüger, Markus Breunig, Lino Pascal Pasquini, Mareen Morawe, Alexander Groß, Frank Arnold, Ronan Russell, Thomas Seufferlein, Ninel Azoitei, Hans A. Kestler, Cécile Julier, Sandra Heller, Meike Hohwieler, Alexander Kleger

**Affiliations:** 1 Department of Internal Medicine I, Ulm University Hospital, 89081 Ulm, Germany; jana.krueger@uni-ulm.de (J.K.); markus.breunig@uni-ulm.de (M.B.); lino.pasquini@uni-ulm.de (L.P.P.); mareen.morawe@uni-ulm.de (M.M.); frank.arnold@uni-ulm.de (F.A.); ronan.russell@gmail.com (R.R.); thomas.seufferlein@uniklinik-ulm.de (T.S.); ninel.azoitei@uni-ulm.de (N.A.); sandra.heller@uni-ulm.de (S.H.); meike.hohwieler@uni-ulm.de (M.H.); 2 Institute of Medical Systems Biology, Ulm University, 89069 Ulm, Germany; alexander.gross@uni-ulm.de (A.G.); hans.kestler@uni-ulm.de (H.A.K.); 3 Cochin, INSERM U1016, CNRS UMR-8104, Université de Paris, 75014 Paris, France; cecile.julier@inserm.fr; 4 Core Facility Organoids, Ulm University, 89081 Ulm, Germany

**Keywords:** stem cells, pancreatic development, definitive endoderm, shRNA

## Abstract

Human pluripotent stem cells, with their ability to proliferate indefinitely and to differentiate into virtually all cell types of the human body, provide a novel resource to study human development and to implement relevant disease models. Here, we employed a human pancreatic differentiation platform complemented with an shRNA screen in human pluripotent stem cells (PSCs) to identify potential drivers of early endoderm and pancreatic development. Deep sequencing followed by abundancy ranking pinpointed six top hit genes potentially associated with either improved or impaired endodermal differentiation, which were selected for functional validation in CRISPR-Cas9 mediated knockout (KO) lines. Upon endoderm differentiation (DE), particularly the loss of SLC22A1 and DSC2 led to impaired differentiation efficiency into CXCR4/KIT-positive DE cells. qPCR analysis also revealed changes in differentiation markers CXCR4, FOXA2, SOX17, and GATA6. Further differentiation of PSCs to the pancreatic progenitor (PP) stage resulted in a decreased proportion of PDX1/NKX6-1-positive cells in SLC22A1 KO lines, and in DSC2 KO lines when differentiated under specific culture conditions. Taken together, our study reveals novel genes with potential roles in early endodermal development.

## 1. Introduction

Human development is a highly complex and tightly regulated process involving a variety of signaling pathways [1] and gene regulatory networks that contribute to differentiation from one totipotent stem cell to fully assembled tissues and organs. The zygote resulting from sperm and oocyte fusion goes through multiple rounds of mitotic division to form a blastocyst. The blastocyst comprises two lineages, the embryoblast or inner cell mass (ICM) and the trophoblast. The ICM undergoes further differentiation and lineage segregation, giving rise to the three germ layers of ectoderm, mesoderm, and definitive endoderm [2,3,4,5], which subsequently generate all mature tissue organs of the body. The definitive endoderm (DE) subsequently forms, besides other organs, the respiratory and gastrointestinal tract, and their derived tissues and organs. Defects in endoderm-derived organs are associated with numerous medical conditions such as liver diseases and diabetes, which affect millions of people every year [5]. Initial studies in mice postulated a widely conserved molecular control of DE development across various species, but recent work revealed that key molecular aspects of endoderm regulation differ between rodents and humans [6,7,8]. Unfortunately, studies reporting on human endoderm development remain sparse, mostly due to the limited availability of time-resolved human embryonic and fetal material [9,10,11]. To bridge this gap, human pluripotent stem cells (PSCs) established from human blastocysts as embryonic stem cells or reprogrammed from human somatic progeny as induced pluripotent stem cells are successfully employed in disease modeling, drug screening, and regenerative medicine [5,12,13]. Various protocols describe the differentiation of such pluripotent stem cells towards the endodermal lineage as well as its derived cell types, such as intestinal cells [14,15,16], hepatocytes [17], pancreatic progenitor cells [18,19,20], or even insulin-producing β-cells [21,22,23]. However, the precise mechanisms governing such complex processes are still only partly understood, and many protocols yield impure populations of the desired cell type. Functional genomic screening using gain- and loss-of-function technologies was successfully used to identify gene networks governing human embryonic stem cell behavior [24]. Our own group recently reported Dickkopf-3 (DKK3) as a novel factor for organ regeneration using combined transcription-factor-induced reprogramming and RNA interference techniques [25]. However, RNA interference screening during pancreatic endoderm formation has not been explored. To dissect the molecular processes occurring during early endodermal and pancreatic differentiation, we identified and characterized new genetic players either promoting or suppressing hESC differentiation into definitive endoderm following an shRNA-based functional genomics approach.

## 2. Materials and Methods

### 2.1. shRNA Screen

HUES8 cells were infected with SFLV-pGipz-GFP-HEL viruses (Thermo Scientific, Waltham, MA, USA) featuring shRNAs based on the Open Biosystems shRNA library [26]. We selected 496 specific shRNAs targeting genes upregulated in either the endoderm or the mesoderm/ectoderm in mice on E7 on the basis of previously published transcriptome data of Pdx1-GFP mice. Cells were differentiated towards definitive endoderm as previously described [18] before being FACS-sorted for either CXCR4^+^/KIT^+^/GFP^+^ endodermal cells or CXCR4-/KIT-/GFP+ nonendodermal cells. DNA was extracted from both cell populations using the Blood and Tissue DNA Kit (Qiagen, Hilden, Germany). Barcode shRNA amplification and sequencing was followed by a comparative analysis of shRNA abundance. Genes were only considered as “Hit” when they were under-represented in one and enriched in the other sample group.

### 2.2. Stem Cell Culture

Human embryonic stem cell line 8 (HUES8) was obtained from Harvard University (RRID:CVCL_B207) and was used with the permission of the Robert Koch Institute according to “79. Genehmigung nach dem Stammzellgesetz, AZ 3.04.02/0084”. Cells were cultivated in either mTeSR1 or mTeSR Plus medium (STEMCELL Technologies, Vancouver, BC, Canada) at 5% CO_2_, 5% O_2_, and 37 °C on hESC-qualified Matrigel (Corning, New York, NY, USA)-coated culture plates. Cells were split twice a week. Briefly, cells were washed with PBS (Gibco, Waltham, MA, USA) and incubated with TrypLE express (Gibco, Waltham, MA, USA) at 37 °C until detachment from the plate. Cells were then resuspended in DMEM-F12+GlutaMAX (Gibco, Waltham, MA, USA), and centrifuged at 210× *g* for 4 min. After resuspension in mTesR medium containing 10 µM ROCK inhibitor (Y-27632; Abcam, Cambridge, UK), cells were seeded again on hESC Matrigel-coated plates.

### 2.3. Generation of Knockout Cell Lines

In total, 50,000 HUES8 cells per well were seeded on Matrigel-coated 24-well plates. Medium was changed to mTeSR with 1× CloneR (Stemcell Technologies, Vancouver, BC, Canada) after 24 h. For KO generation, 3 pmol of Cas9 enzyme and 18 pmol of the respective gRNA (Synthego, Menlo Park, CA, USA) were combined in 25 µL DMEM-F12 (Gibco, Waltham, MA, USA) and incubated for 15 min at RT for RnP complex formation. Then, 25 µL of DMEM-F12 containing 1 µL Lipofectamine Stem Reagent (Invitrogen, Waltham, MA, USA) was added to the mix and incubated for another 15 min at RT before dropwise delivery to the cells. After 24 h, the medium was replaced with fresh mTeSR with CloneR. Cells were harvested after 48 h using TrypLE and then filtered through a 40 µm filter. Then, 100, 200, or 300 cells were seeded in mTeSR with CloneR on Matrigel-coated 6-well plates for clonal expansion. After approximately 10 days, single-cell-derived colonies were manually scraped from the plate. Half of each colony was used for DNA isolation and sequencing analysis, while the other half was kept in culture for further investigations. Clones that harbored a KO in the respective genes, and CRISPR WT control lines were expanded for further experiments. The following gRNA sequences were used for the different target genes: ACCAACACAGACACGAAGGU (*DKK3*), UAUAGUUCAGCUCCUCCGCA (*SLC22A1*), GCUGUCAAGAGCCUUCCUGC (*CSRP3*), UGACACCUUGUAACCCCGGG (*PMM1*), GGGUCCCGUCCACGUCAAAC (*DSC2*), UUUCUCGGCAUCUAGUUUGG (*COL4A1*).

### 2.4. DNA Isolation, PCR and Sequencing

The picked colonies were subjected to DNA isolation using the Blood DNA Purification Mini Prep Kit (Genaxxon, Ulm, Germany) according to manufacturer instructions. The genes of interest were amplified using GoTaq polymerase (Thermo, Waltham, MA, USA), and PCR products were purified using the Wizard^®^ SV Gel and PCR Clean-Up System (Promega, Madison, WI, USA), before being sent to Eurofins Genomics for sequencing. The genotype of clones was verified using the ICE analysis online tool from Synthego. For PCR amplification, the following primer sequences were used: ACCCACCTCCCAGAGAGATT and TCCTTTGCAACTGGACTGGC for *DKK3*, CATGCTGAGCCATCATGCCC and AGCCAGACACCCACGAACTG for *SLC22A1*, GGGATGCAGTCCTTAGCAGG and TTTAACAGGCAAGGGGGAGC for *CSRP3*, CTTCTGCCGTTGCATCTTCG and GACCACTGGTGTGTCGTAGG for *PMM1*, TCTCCCCACGTGCATACATT and CACTGTGAAGTTGCCTCATGG for *DSC2* and CCTTGACTCAGGCAGTGGACT and CCAGGAGTCTCAGAGGTGGTT for *COL4A1*, respectively. For PCR, GoTaq buffer was mixed with 1.5 mM MgCl_2_, 0.5 pM of both forward and reverse primer, 5 mM dNTPs and 50–100 ng of template DNA. Initial denaturation was performed for 5 min at 95 °C, followed by 35 cycles of 45 s denaturation at 95 °C, 30 s annealing at 60–63 °C, and 1 min elongation at 72 °C. Amplification was terminated after a final elongation step of 10 min at 72 °C.

### 2.5. Differentiation into Pancreatic Progenitor Cells

To drive differentiation of hPSCs into pancreatic progenitor cells (PPs), a protocol originally published by Nostro and colleagues [27] and optimized by our group [18,28] was employed. Briefly, 300,000 cells were seeded per 24-well in mTesR1 medium supplemented with 10 µM ROCK inhibitor in wells precoated with 0.5 mg/mL GFR-Matrigel (Corning, New York, NY, USA). Differentiation was started after 24 h at 80–90% cell confluency by adding day 0 differentiation medium. For a detailed description of media composition and cytokines, please see Breunig et al. [18,28]. Cells were incubated in 5% CO_2_ atmosphere at 37 °C and the medium was changed daily. Samples were harvested for flow cytometry analysis or RNA isolation at day 3 (definitive endoderm), day 9 (pancreatic endoderm), and day 13 (pancreatic progenitor).

### 2.6. RNA Isolation and qPCR

For qPCR analysis, cells were harvested with TrypLE express as described above. After washing with PBS, cell lysis and RNA isolation were performed using the GeneJET RNA Purification Kit (Thermo, Waltham, MA, USA). RNA was transcribed to cDNA with the iScript cDNA Synthesis Kit (Bio-Rad, Hercules, CA, USA), and qPCR was performed using the SensiMix SYBR^®^ No Rox Kit (Bioline, London, UK) and measured with a Quantstudio 3 cycler (Thermo Fisher, Waltham, MA, USA) according to manufacturer’s instructions. Quantitect primers were obtained from Qiagen (Hilden, Germany) (QT00223188 for *CXCR4*, QT00204099 for *SOX17*, QT01864940 for *GATA6* and QT00212786 for *FOXA2*, QT00036057 for *DKK3*, QT00019572 for *SLC22A1*, QT00013146 for *PMM1*, QT00016128 for *DSC2*, QT00005250 for *COL4A1*) or self-designed and ordered from Biomers (Ulm, Germany) (*CSRP3* forward GCTCAGTTACCACCAGCAACC, reverse AACCTTCTCAGCAGCATAGACT).

### 2.7. Flow Cytometry

Cells were harvested using TrypLE at hESC, DE, PE, and PP stages as described above. At the hESC and DE stages, living cells were used for surface-marker staining, whereas cells at the hESC, PE, and PP stages were sampled for intracellular staining. DE cells were blocked in 10% FCS in PBS for 30 min on ice before incubation in blocking solution containing the anti-CXCR4-PE antibody (Thermo (Waltham, MA, USA), MHCXCR404, 1:50) for 40 min on ice in the dark. Subsequently, cells were incubated with the KIT-APC antibody (Thermo (Waltham, MA, USA), CD11705,1:100) for another 10 min. Cells were washed twice with 2% FCS in PBS before being filtered and subjected to LSR II flow cytometry (BD, Franklin Lakes, NJ, USA). 150 ng/mL DAPI was added before measuring to exclude dead cells from the measurement. For hESC surface marker staining, cells were also blocked as described above, but were then incubated with TRA1-60-FITC (BD (Franklin Lakes, NJ, USA), 560128, 1:10) and SSEA4-PE antibodies (BD (Franklin Lakes, NJ, USA), MC813-70, 1:10) for 1 h on ice in the dark before being washed and measured as described for DE samples.

For intracellular flow cytometry, cells in the hESC, PE and PP stages were fixed in 4% PFA (Sigma-Aldrich, St. Louis, MO, USA) with 100 nm sucrose (Sigma-Aldrich, St. Louis, MO, USA) in PBS for 25 min on ice, washed with PBS and incubated in blocking solution (5% normal donkey serum (NDS, Jackson ImmunoResearch, Cambridgeshire, UK) in 0.1% Triton-X/PBS) for 30 min on ice. After centrifugation at 900× *g* for 3 min, primary antibodies diluted in blocking solution were added to the cells and incubated overnight at 4 °C. For flow cytometry analysis, SOX2 (R&D (Minneapolis, MN, USA), MAB2018, 1:300), NANOG (Cell signaling (Danvers, MA, USA), 3580, 1:100), OCT4 (Santa Cruz (Dallas, TX, USA), sc-8628, 1:500), PDX1 (R&D (Minneapolis, MN, USA), AF2419, 1:500) and NKX6-1 (DSHB (Iowa City, IA, USA), F55A12-concentrate, 1:125) antibodies were used. After washing twice with washing solution (2% NDS in 0.1% Triton-X/PBS), cells were incubated in secondary antibodies (Alexa Fluor, Thermo (Waltham, MA, USA), 1:500) diluted in blocking solution for 1.5 h on ice in the dark before being washed with washing solution, filtered, and measured.

### 2.8. ICC Staining

For analysis of the pluripotency status of the newly generated cell lines, cells were seeded on Matrigel-coated 24-well ibiTreat µ-plates (ibidi, Gräfeling, Germany) and cultivated until 90% confluent. Cells were fixed in 4% PFA with 100 nm sucrose in 0.1% Triton-X/PBS for 20 min at RT. After washing with PBS, 50 mM NH_4_Cl (Sigma-Aldrich, St. Louis, MO, USA) was added for quenching for 10 min, followed by additional washing steps. Permeabilization and blocking were conducted in blocking solution (5% normal donkey serum in 0.1% Triton-X/PBS) for 30 min. Incubation with primary antibodies NANOG (Cell Signaling (Danvers, MA, USA), 3580. 1:100) and OCT4 (Santa Cruz (Dallas, TX, USA), sc-5279, 1:200) was performed at 4 °C, followed by washing with washing solution (2% NDS in 0.1% Triton-X/PBS) and incubation with secondary antibodies (Alexa Fluor, Thermo (Waltham, MA, USA), 1:500) and 500 ng/mL DAPI diluted in blocking solution for 1.5 h. Cells were subjected to a final wash with PBS before imaging with a BZ-9000 fluorescence microscope (Keyence, Osaka, Japan).

### 2.9. Bioinformatics Analysis

Analysis was carried out using DESeq 1.18.1 under R 3.4.4. as previously described [29]. R version 3.4.4 (2018-03-15), platform: x86_64-pc-linux-gnu (64-bit), running under Ubuntu 18.04.6 LTS.

### 2.10. Statistical Analysis

Statistical analysis was performed using GraphPad Prism (Graphpad, San Diego, CA, USA). For all comparisons between KO and WT, the Mann–Whitney test was used. For RNA sequencing data, one-way ANOVA tests were applied. The level of significance is indicated in the graphs as follows: *** *p* < 0.001, ** *p* < 0.01, * *p* < 0.05.

## 3. Results

### 3.1. RNA Interference Approach during Endodermal Differentiation of Human PSCs

**Figure 1:**
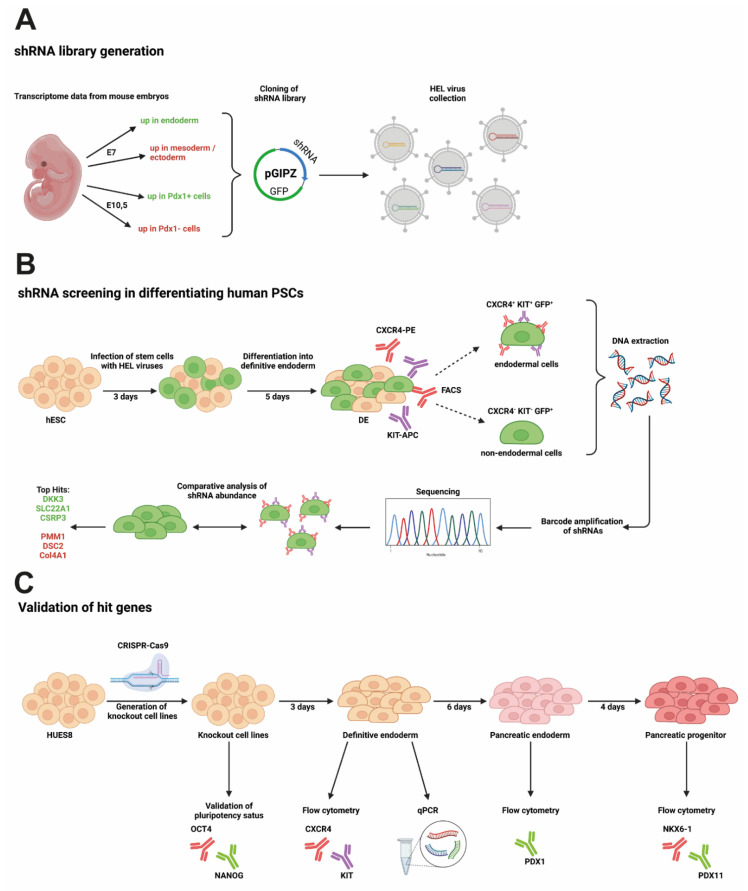
Workflow of genomic screen to identify players in endoderm formation. Schematic illustrating (**A**) workflow from shRNA library generation via (**B**) shRNA screening in hES cells driven towards definitive endoderm to (**C**) validation of hit genes by knockout cell line generation and differentiation. Created with BioRender.com.

**Figure 2:**
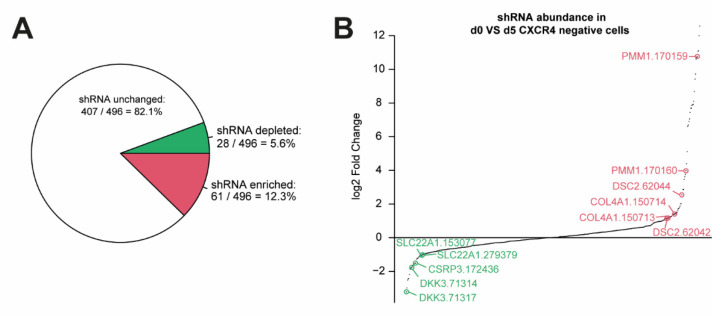
6 hit genes were discovered in an shRNA screen in early endoderm development. (**A**) Pie chart highlighting numbers and shares of enriched (red), depleted (green), or unchanged shRNAs in CXCR4 negative cells vs. undifferentiated d0 cells. Criteria for a significant change were a minimal absolute log2 fold change of 1 (corresponding to either an increase by 100% or a decrease by 50%) and a maximal FDR-adjusted *p*-value of 0.1. (**B**) Log2 fold changes in shRNA abundance of d0 cells vs. CXCR4 negative cells after differentiation to DE stage. Selected sequences are highlighted.

**Figure 3:**
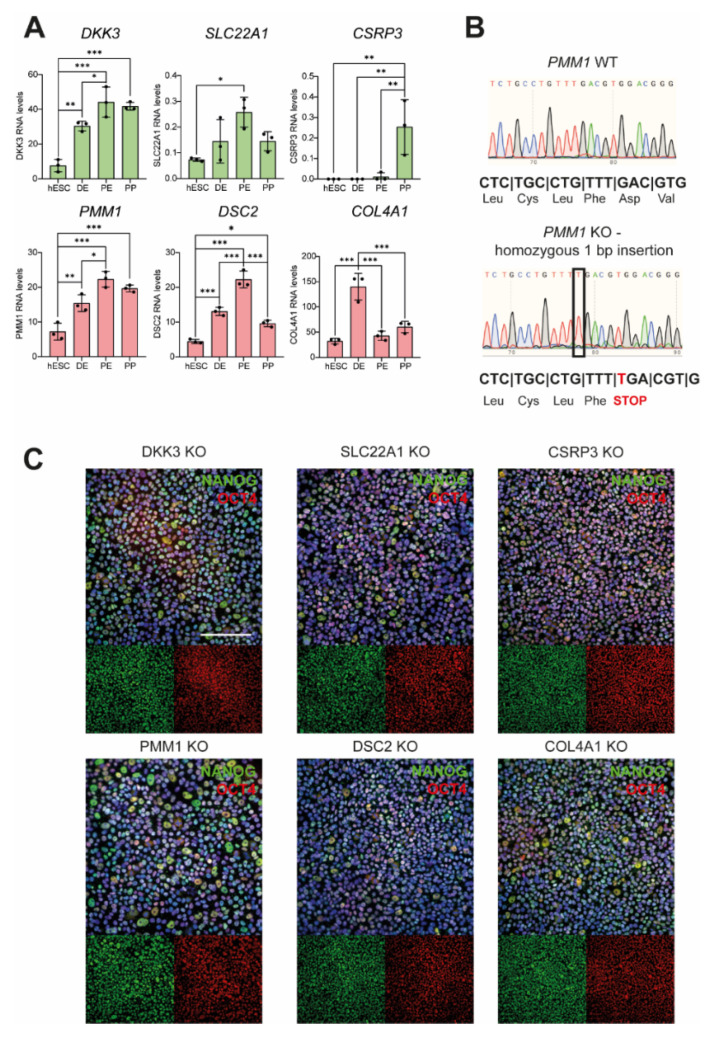
KO of hit genes does not impair pluripotency marker expression. (**A**) Expression of top six genes identified in the shRNA screen was analysed in previously published RNA sequencing data [30]. Expression levels shown for human embryonic stem cell (hESC), definitive endoderm (DE), pancreatic endoderm (PE), and pancreatic endoderm (PP) stages (n = 3 independent experiments in technical duplicates, dots represent means of respective duplicates). One-way ANOVA tests were used to calculate statistical significances. Only significant differences are indicated. Error bars represent mean ± SD, *** *p* < 0.001, ** *p* < 0.01, * *p* < 0.05. (**B**) Successful KO of target genes was validated using PCR amplification and sequencing of the suspected gRNA–Cas9 complex target site. (**C**) Generated KO cell lines were analyzed for the expression of pluripotency factors NANOG (green) and OCT4 (red). Nuclei are stained with DAPI in blue. Scale bar represents 100 µm. Representative images of one clone per genotype are shown.

An RNA interference (RNAi) screen was used to identify gene knockdowns that enhance or limit early endodermal and pancreatic differentiation in human PSCs. The customized and focused human endoderm library (HEL) was compiled from a genomewide shRNA library on the basis of public time-resolved transcriptome data derived from PDX1-GFP reporter mice at embryonic days E7 and E10.5. In total, we selected 496 shRNA constructs targeting human genes that had been upregulated in either the endoderm or the mesoderm/ectoderm, or Pdx1+ or Pdx1− cells, respectively (Figure 1A). The HEL shRNA library carrying a GFP label was introduced into human PSC line HUES8 by lentiviral delivery, and cells were differentiated to the DE stage. To lower the risk of multiple infections of the same cell, the amount of virus was adjusted to achieve infection rates of around 40%. FACS-based sorting for either CXCR4+/KIT+/GFP+ endodermal cells or CXCR4-/KIT-/GFP+ nonendodermal cells, and deep sequencing was employed to determine the abundance of shRNAs in both populations (Figure 1B). The abundance of shRNAs was then compared to the abundance obtained directly after viral transduction at day 0 (Supplementary Tables S1 and S2). Genes were considered to be a “hit” either when they were under-represented in the CXCR4-positive and enriched in the CXCR4-negative cell population (or vice versa) or if multiple shRNAs targeting the same gene were enriched or depleted in one group (Figure 2A). Only six genes matched these criteria and were selected for further investigation (Figure 2B,C). The knockdown of three genes resulted in increased DE formation in the initial screen: Dickkopf-3 (*DKK3*), solute carrier family 22 member 1 (*SLC22A1*), and cysteine and glycine rich protein 3 (*CSRP3*). The knockdown of the other three genes (i.e., phosphomannomutase 1 (*PMM1*), desmocollin 2 (*DSC2*), and collagen type iv alpha 1 chain (*COL4A1*) led to decreased DE formation (Figure 2A,B). Next, we consulted our previously reported RNA sequencing data obtained during stage-specific pancreatic differentiation of human PSCs [30,31] to decipher gene regulation patterns of the respective hits: the expression of *DKK3*, *SLC22A1*, *PMM1*, and *DSC2* increased throughout differentiation with a peak at the pancreatic endoderm (PE) stage, followed by a lower expression at the pancreatic progenitor (PP) stage (Figure 3A). *COL4A1*, on the other hand, showed a peak of expression at DE stage with lower expression levels throughout the other stages, and *CSRP3* expression was only detected from the PE stage, with a peak at the PP stage.

### 3.2. Newly Generated Knockout Cell Lines Still Express Pluripotency Markers

To thoroughly validate these hit genes, CRISPR-Cas9 technology was employed to establish homozygous knockout (KO) PSC lines for all six hits. gRNAs were designed to bind at the beginning of each locus, thereby leading to double-strand breaks and repair by nonhomologous end joining often resulting in insertions or deletions. The exact mutation of the edited clones was determined by Sanger sequencing and only clones in which the mutation was predicted to result in a premature stop codon in the target gene were selected (exemplarily shown for *PMM1* in Figure 3B; all other sequences are shown in Supplementary Figures S1 and S2; insertions and deletions of all clones are listed in Table 1). Two clonal KO lines were selected for each gene. Wild-type (WT) clones that harbored no aberrant bases in the targeted region, but had undergone the same gene editing and clonal expansion procedure were used as controls in subsequent differentiation experiments. Reduced mRNA levels of the respective genes were observed upon qPCR analysis in some KO clones, most likely due to reduced RNA stability (Supplementary Figure S3A). Since the premature stop codon still allowed for the transcription of the altered genes, such trend was not present throughout all hit genes. After the expansion of the clones, the genotype of all single-cell-derived clonal lines was again confirmed by sequencing. To verify pluripotency, the expression of pluripotency markers NANOG [32] and OCT4 [33] was verified by immunofluorescence staining in all tested KO clones (exemplarily shown for one clone per genotype in Figure 3C). No obvious differences in morphology of the cells were observed. Additionally, flow cytometry staining was performed, validating results obtained for NANOG and OCT4. Furthermore, all cell lines were found to express stem cell markers TRA1-60, SSEA4, and SOX2 (Supplementary Figure S1B) indicating preserved pluripotency in all KO lines.

### 3.3. Knockout of DSC2 and SLC22A1 Leads to Impaired DE Formation

**Figure 4:**
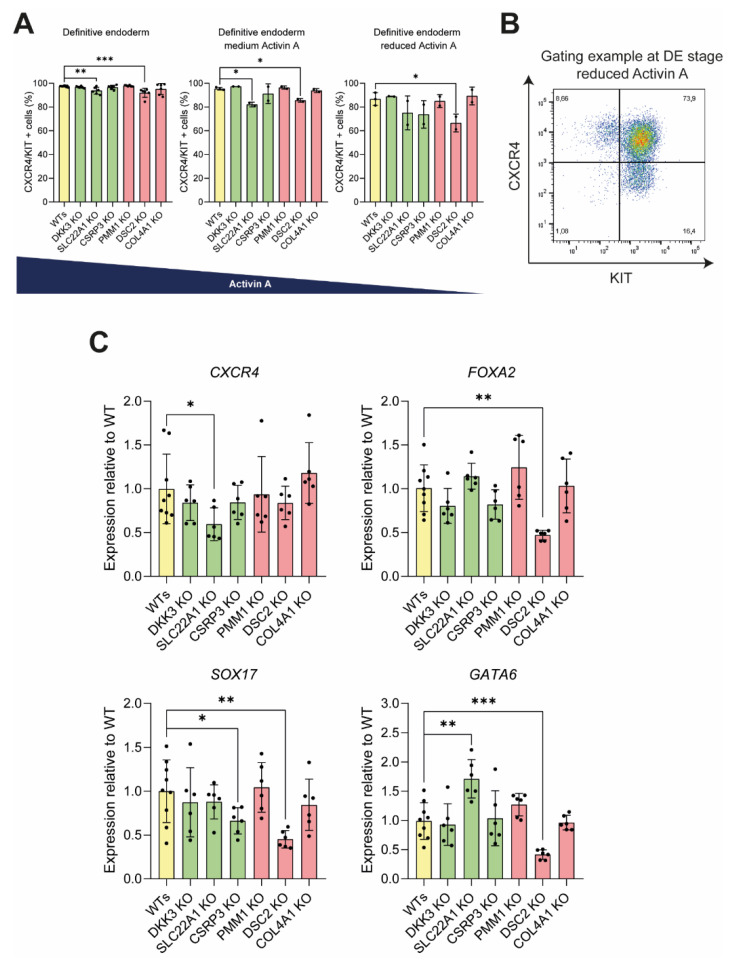
DSC2 and SLC22A1 KO lines show an impaired differentiation into definitive endoderm. (**A**) Newly generated KO cell lines were differentiated towards DE stage with either the standard induction protocol (left), or with only 10% or 5% (right) of standard activin A concentration. (**B**) Differentiation efficiency was analysed by flow cytometry of DE markers CXCR4 and KIT, as shown in a representative FACS plot. (**C**) From cells generated under optimal culture conditions, RNA samples were taken and analysed via qPCR for expression levels of DE markers *CXCR4*, *FOXA2*, *SOX17,* and *GATA6*. Experiments with standard culture conditions were performed 3 times, using 2 different clones per genotype in technical duplicates (dots represent means of duplicates). Experiments with reduced levels of activin A were performed once with 2 different clones per genotype and in duplicates (dots represent means of these duplicates). Gene expression was first normalized to housekeeping gene HMBS and then normalized to WT gene expression. Mann–Whitney test was used for analysis; error bars represent mean ± SD, *** *p* < 0.001, ** *p* < 0.01, * *p* < 0.05.

Next KO PSC lines were directed towards DE stage following our previously published differentiation protocol [18,20,28,34]. The efficiency of lineage commitment was validated by analyzing the expression of endodermal markers CXCR4 [33,35] and KIT [36] via flow cytometry. The lack of SLC22A1 or DSC2 led to a significant decrease in DE formation when compared to WT control cell lines (Figure 4A,B). The difference was further accentuated upon a reduction of activin A in the culture medium, a potent endoderm formation cytokine [14,37] (Figure 4A,B). While the initial screen loss of DSC2 was predicted to impair endoderm formation, the loss of SLC22A1 was hypothesized to cause the opposite effect. The loss of the four other genes did not lead to any significant increase or decrease in CXCR4/KIT-positive cells at the DE stage. Further analysis of DE markers on mRNA level revealed a significant decrease in endoderm markers *FOXA2* [38], *SOX17* [39], and *GATA6* [5,39,40] in the *DSC2* KO lines (Figure 4C). *SLC22A1* KO lines exhibited a significantly reduced expression of *CXCR4*, whereas *GATA6* expression was significantly increased, thereby suggesting an aberrantly altered DE composition. In CSRP3 KO lines, *SOX17* levels were decreased. All other KO cell lines did not differ significantly in DE marker expression.

### 3.4. Pancreatic Progenitor Formation Is Affected by DSC2 and SLC22A1 Loss

**Figure 5:**
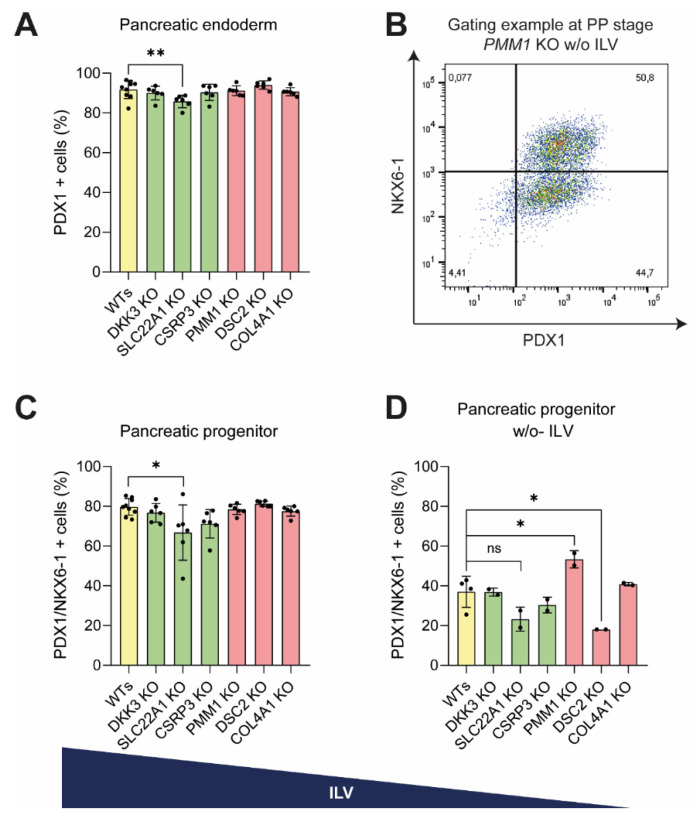
PP formation is affected by the loss of DSC2 and SLC22A1. KO cell lines were differentiated to (**A**) pancreatic endoderm stage followed by (**B**–**D**) pancreatic progenitor stage. Differentiation efficiency was analysed by flow cytometry staining of PDX1 (PE) or PDX1 and NKX6-1 (PP) (n = 3 independent experiments, 2 different clones per genotype and 3 different WT clones, each clone in technical duplicates, dots represent means of these duplicates). (**B**,**D**) To better unveil differences in differentiation efficiencies, cells were differentiated without indolactam V (ILV) (n = 1 experiment, at least 2 clones per genotype each in technical duplicates). (**B**) Representative FACS plot. Mann–Whitney test was used for statistical analysis; error bars represent mean ± SD, ** *p* < 0.01, * *p* < 0.05.

To investigate whether the ablation of our candidate genes might also be relevant in further maturation into pancreatic cells, DE cells were further differentiated into pancreatic endoderm (PE) and pancreatic progenitors (PPs). With a protocol rendering 95% PDX1-positive PE and 80% PDX1/NKX6-1 positive PP cells under optimal conditions, only the loss of SLC22A1 resulted in a small but significant reduction in PE and PP formation. (Figure 5A,C). At the same time, all the other KO cell lines differentiated with efficiencies similar to the WT control lines (Figure 5A–C). However, a reduction in differentiation efficiency upon omitting the PKC activator indolactam V from the induction medium [30] revealed a reduced proportion of PPs for SLC22A1 and DSC2 KO lines (Figure 5D), in line with the differences already observed at the DE stage. Surprisingly, the KO of PMM1 led to an increase in differentiation efficiency of about 20% compared to WT cell lines. Taken together, out of the six investigated hits, only SLC22A1- and DSC2-deficient hESCs exhibited significant impairment in DE and PP differentiation. Further investigations could help to elucidate the exact role for development and disease.

## 4. Discussion

We conducted a functional, customized, and focused genomics screen in human pluripotent stem cells undergoing lineage specification into definitive endoderm to reveal potential genetic players acting during early development. KO cell lines were generated using CRISPR-Cas9 technology in order to validate the top six hit genes. While four of the hits could not be verified through follow-up experiments, our findings revealed decreased differentiation efficiency for two of the investigated candidates: *DSC2* and *SLC22A1*.

DSC2 is a member of the desmocollin protein subfamily comprising cadherin-like transmembrane glycoproteins. Together with the desmogleins, they form the desmosomes, cell–cell junction proteins found in tissue experiencing mechanical stress such as the heart, respiratory tract, bladder tissue, or the gastrointestinal mucosa [41], many of which derived from the endoderm, but also in epithelial cells of the pancreas. *DSC2* mutations cause arrhythmogenic cardiomyopathy, generally under autosomal dominant inheritance [42,43] and autosomal recessive in a minority of cases [44]. In addition, the reduced expression of *DSC2* was reported in different types of cancer [45,46]. Patients with biallelic *DSC2* mutations may present additional clinical features to heart defects, including skin and hair abnormalities [47].

Since desmosomes fulfill the function of a signaling hub in the cell orchestrating cellular processes such as cell–cell adhesion [48], proliferation, or migration, we envisaged a potentially important role for development and differentiation. While the loss of DSC2 resulted in a moderate decrease in DE formation, PP differentiation was only impaired under specific culture conditions. Hence, albeit DSC2 was not essential for endoderm and pancreatic progenitor formation, perturbations in its expression and function might perturb the gene regulatory network involved in endodermal organ formation. The loss of DSC2 also resulted in a significant reduction in GATA6 expression at the endoderm stage, a marker vital for endoderm induction [6] that had been associated with the development of diabetes [49], pancreatic insufficiency, and congenital heart disease [50], and which plays a vital role in insulin production in adult β-cells [51]. Although pancreatic disorders or diabetes are not recognized characteristic features of patients with *DSC2* mutations, a case of nonautoimmune diabetes diagnosed as type 2 diabetes was reported in one of the few described patients with biallelic *DSC2* deficiency [44]. Whether diabetes is a coincidental finding in this patient or a possible feature of complete *DSC2* deficiency remains to be established.

While the loss of SLC22A1, an organic cation transporter 1 (OCT1) protein, also seemed to negatively impact DE formation, as demonstrated by lower levels of CXCR4 and KIT, the expression of endodermal marker GATA6 was actually increased in KO lines, while other investigated markers Sox17 and FOXA2 remained unchanged. The original shRNA screen indicated that loss of SLC22A1 rather promoted cell differentiation towards DE. A possible explanation for the discrepancies between shRNA and CRISPR approach might be that the remnant expression after shRNA knockdown could suffice for various cellular processes, while a complete loss of the protein results in an impairment of differentiation, suggesting a presumably tightly regulated dose-dependent role of SLC22A1 during endodermal development.

SLC22A1 is a transmembrane protein working as a polyspecific organic cation transporter, mainly in the liver [52]. So far, it has mainly been investigated in the context of different cancer entities, such as hepatocellular carcinoma, where it mediates the uptake of cationic chemotherapeutics [53,54]. The association of SLC22A1 variants with adverse gastrointestinal response of T2D patients to metformin was also reported [55,56]. A potential role of this protein in early development, however, has not yet been described. We hypothesize that developmental defects of the liver and/or the endocrine pancreas, indicated by decreased in vitro DE and PP formation after ablation of *SLC22A1*, might contribute to this adverse response to metformin treatment in T2D patients carrying reduced-function *SLC22A1* variants. Although developmental defects are likely not the main driver, further investigations could help to investigate if they contribute to the pathomechanism of adverse metformin effects in such patients.

In summary, our shRNA screen involving embryonic stem cells differentiated to the definitive endoderm stage rendered a list of hit genes. Analysis of the six most promising candidates revealed a potential role in endoderm development for two of the hits, where one hit was associated with altered glycemic control in patients with type 2 diabetes. However, to understand the role of DSC2 and SLC22A1 in early development and pathological manifestations in more detail, further studies need to be conducted.

## Figures and Tables

**Table 1:** Indels leading to premature stop codons in hit genes.

Gene	Clone	Allele 1	Allele 2
DKK3	1	+1 bp insertion	+1 bp insertion
	2	+1 bp insertion	−2 bp deletion
SLC22A1	1	−1 bp deletion	−8 bp deletion
	2	+1 bp insertion	−1 bp deletion
CSRP3	1	+1 pb insertion	−4 bp deletion, +10 bp insertion
	2	−1 bp deletion	−26 bp deletion
PMM1	1	+1 bp insertion	+1 bp insertion
	2	−7 bp deletion	−8 bp deletion
DSC2	1	−2 bp deletion	−11 bp deletion
	2	−1 bp deletion	−10 bp deletion
COL4A1	1	−1 bp deletion	+1 bp insertion
	2	+1 bp insertion	+1 bp insertion

## Data Availability

Raw data is available upon request.

## Supplementary Materials

The following supporting information can be downloaded at: https://www.mdpi.com/article/10.3390/cells11030582/s1, Figure S1: Sequences of WT and KO clones of DKK3, SLC22A1 and CSRP3; Figure S2: Sequences of WT and KO clones of PMM1, DSC2 and COL4A1; Figure S3: Reduced RNA levels of KO genes are observed via qPCR and pluripotency markers are retained after genome editing; Table S1: List of all hits shown in Figure 2B—d0 vs. d5 CXCR4 negative cells; Table S2: List of all hits—d0 vs. d5 CXCR4 positive cells.
