# Supplementary material for: Functional Genomic Screening in Human Pluripotent Stem Cells Reveals New Roadblocks in Early Pancreatic Endoderm Formation
Source: Cells. 2022 Feb 8;11(3):582. doi: 10.3390/cells11030582 (PMC8834018; doi:10.3390/cells11030582)
Supplement: Supplementary file 1 [file cells-11-00582-s001.zip › cells-1546538-supplementary.pdf]

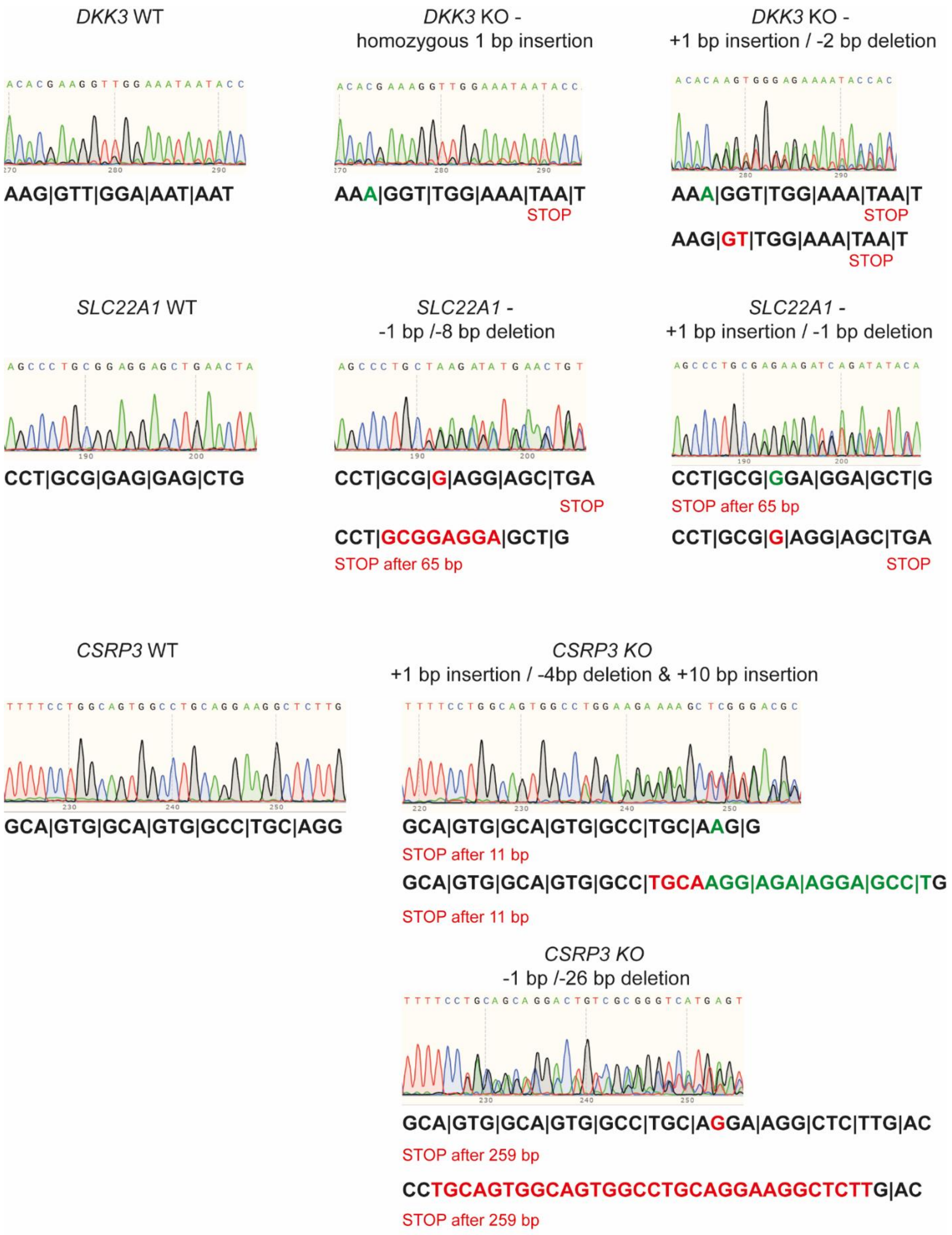

Figure S1: Sequences of WT and KO clones of DKK3, SLC22A1 and CSRP3. KOs were validated using Synthego's "TIDER" online tool and double checked by hand.

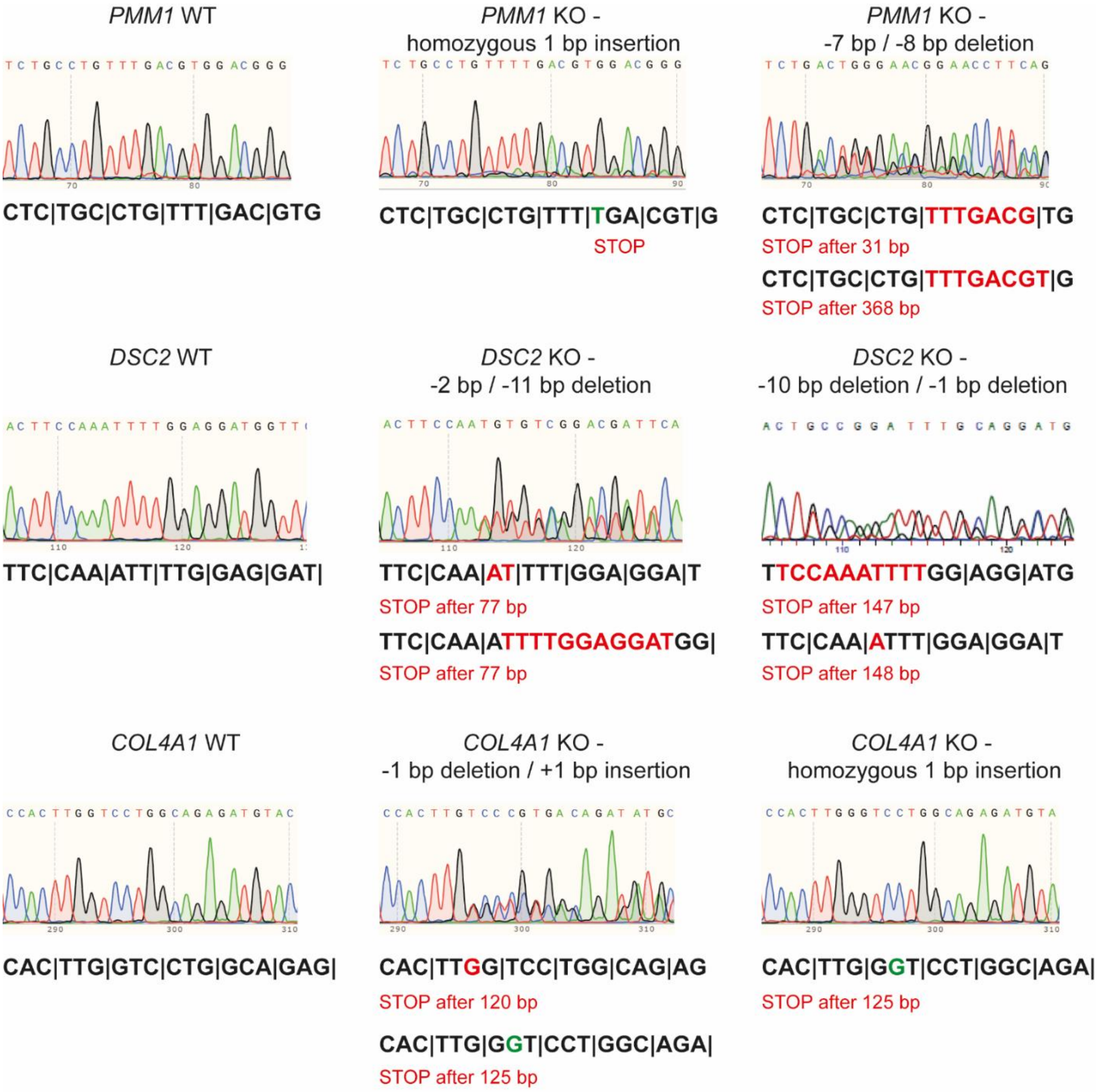

Figure S2: Sequences of WT and KO clones of PMM1, DSC2 and COL4A1. KOs were validated using Synthego's "TIDER" online tool and double checked by hand.

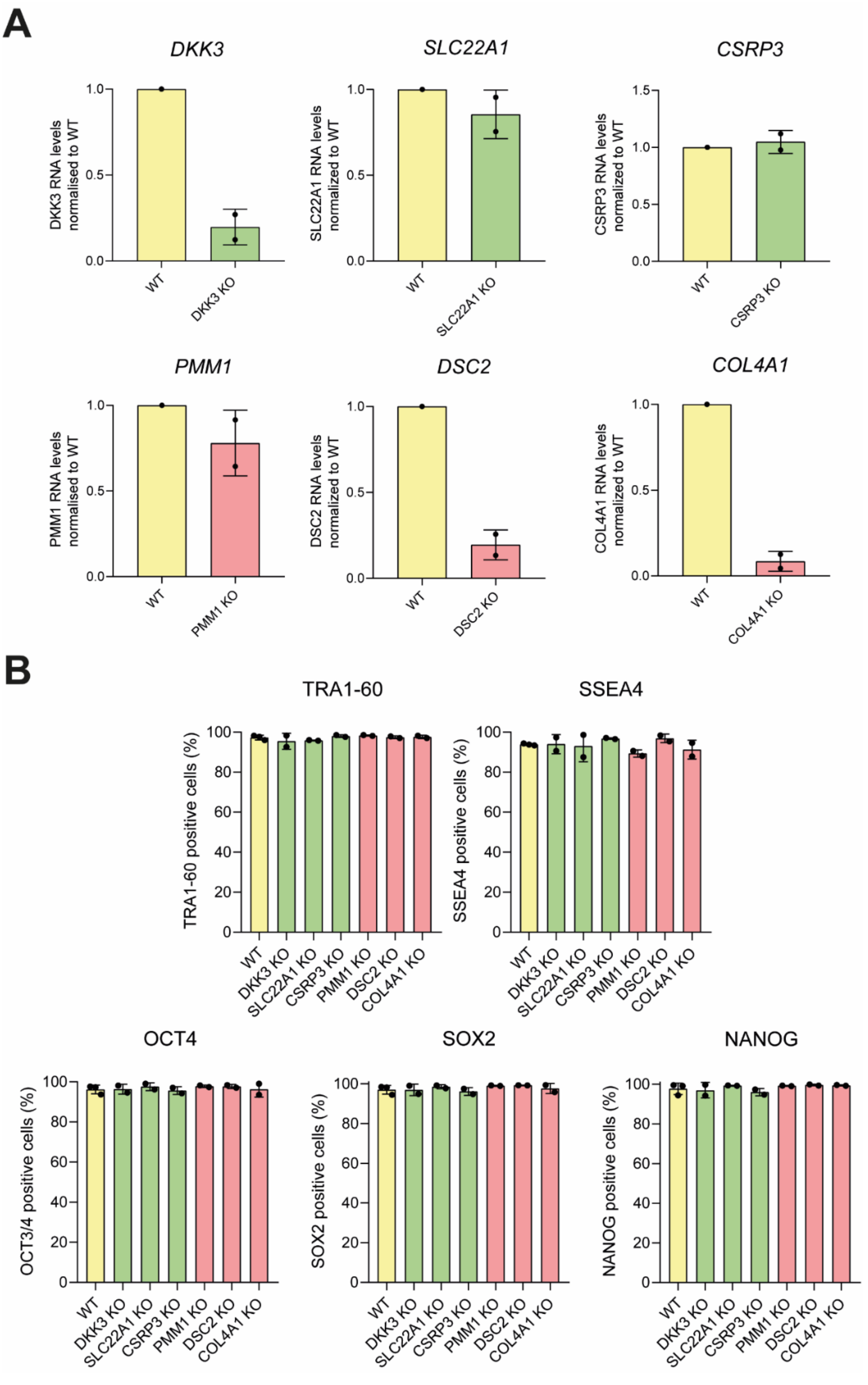

Figure S3: Reduced RNA levels of KO genes are observed via qPCR and pluripotency markers are retained after genome editing. A) qPCR performed with RNA from pancreatic progenitor cells shows a decrease in RNA after gene editing (n=1 experiment, 2 clones per genotype in technical duplicates). B) Expression of pluripotency markers was validated by flow cytometry (n=1 experiment, 2 clones per genotype).

Table S1: List of all hits shown in Figure 2B –
d0 vs. d5 CXCR4 negative cells

Gene	shRNA ID	Log2 fold change
PDCD6	V2LHS_71869	12,569
CPD	V2LHS_113166	11,993
GATM	V2LHS_33446	11,097
ATP5J2	V2LHS_263664	11,085
PMM1	V2LHS_170159 10	10,766
LYN	V2LHS_134144	10,614
JUNB	V2LHS_235957	9,723
STMN4	V2LHS_116885	9,424
Matr3	V2LHS_22224	8,686
USMG5	V2LHS_177355	8,113
AHR	V2LHS_132480	7,921
CLDN7	V2LHS_113182	7,894
HIP2	V2LHS_37619	7,866
DKK1	V2LHS_19944	7,698
TIMM10	V2LHS_43241	7,696
CDKN2B	V2LHS_51831	7,440
APOA4	V2LHS_93246	6,910
PLA2G10	V2LHS_2651	6,699
SLC7A3	V2LHS_177606	6,620
PTGIS	V2LHS_131518	5,111
CPN1	V2LHS_113187	4,398
PMM1	V2LHS_170160 3	3,974
ITM2A	V2LHS_67751	3,749
RBP7	V2LHS_118309	3,625
XPA	V2LHS_92694	3,470
SLC7A7	V2LHS_46447	2,664
DSC2	V2LHS_62044 2	2,546
NR1H3	V2LHS_239181	2,022
PDZK1	V2LHS_169832	2,017
IGFBP6	V2LHS_32798	1,876
SFRS2IP	V2LHS_36802	1,791
NGDN	V2LHS_76266	1,727
VIL1	V2LHS_197376	1,723
MAP3K5	V2LHS_2204	1,641
FOXA2	V2LHS_86206	1,515
BTBD1	V2LHS_196030	1,515
TFPI2	V2LHS_198390	1,433
CA4	V2LHS_112199	1,427
COL4A1	V2LHS_150714 1	1,406
INA	V2LHS_177282	1,396
F10	V2LHS_93365	1,389
RNF7	V2LHS_13708	1,353
LTA	V2LHS_227733	1,350
CITED1	V2LHS_173199	1,276
GLRA1	V2LHS_82866	1,241
FGFBP1	V2LHS_49795	1,223
EPHA2	V2LHS_17961	1,207
ROCK1	V2LHS_70607	1,194
COL4A1	V2LHS_150713 1	1,170

BCHE	V2LHS_259891	1,151
DSC2	V2LHS_62042 1	1,138
CYP26A1	V2LHS_112498	1,133
DAB2	V2LHS_150163	1,130
CHRDL2	V2LHS_136912	1,079
LRP10	V2LHS_59766	1,063
PDCD6IP	V2LHS_64525	1,049
BCAT1	V2LHS_64327	1,035
PRG1	V2LHS_170386	1,031
SLC22A1	V2LHS_153077	-1,012
SLC22A1	V2LHS_279379	-1,041
PREPL	V2LHS_118927	-1,042
DKK3	V2LHS_71314	-1,042
POU1F1	V2LHS_230873	-1,045
DKK3	V2LHS_71317	-1,045
CXCL12	V2LHS_111679	-1,054
PBOV1	V2LHS_200991	-1,082
DIO2	V2LHS_225563	-1,134
NMI	V2LHS_4367	-1,194
PHGDH	V2LHS_91258	-1,214
CETN3	V2LHS_14771	-1,230
CSRP2BP	V2LHS_100125	-1,267
CTSZ	V2LHS_113325	-1,404
CSRP3	V2LHS_172436	-1,508
FOLR1	V2LHS_135471	-1,540
PON2	V2LHS_231553	-1,603
LNX1	V2LHS_159554	-1,609
FHL1	V2LHS_131742	-1,637
SLC16A10	V2LHS_176201	-1,683
LRP8	V2LHS_160292	-1,794
RAE1	V2LHS_27966	-1,867
ABCB4	V2LHS_270743	-2,423
PIGK	V2LHS_68405	-2,442
RRBP1	V2LHS_47386	-2,510
CPA3	V2LHS_150836	-2,983

Table S2: List of all hits –
d0 vs. d5 CXCR4 positive cells

Gene	shRNA ID	Log2 fold change
EPHA8	V2LHS_65935	8,375
DKK3	V2LHS_71317	2,457
GPC1	V2LHS_114228	1,422
CLDN7	V2LHS_113182	1,082
GADD45B	V2LHS_114575	1,063
CSRP3	V2LHS_172436	1,032
PTGIS	V2LHS_131518	-1,119
Matr3	V2LHS_22224	-1,297
ZFP37	V2LHS_172098	-1,340
PDZK1	V2LHS_169832	-1,435
CDKN2B	V2LHS_51831	-1,768
PMM1	V2LHS_170159	-1,969
SCEL	V2LHS_16720	-1,999
HIP2	V2LHS_37619	-2,994
USMG5	V2LHS_177355	-4,689
DKK1	V2LHS_19944	-6,195
CKB	V2LHS_150588	-6,211
